# Driving the Consumer Adoption of Halal Cosmetics: A Systematic Review Using PRISMA and TCCM Framework

**DOI:** 10.1111/jocd.70479

**Published:** 2025-10-17

**Authors:** Md Wasim Raza, Furquan Uddin, Osmud Rahman, Md Billal Hossain

**Affiliations:** ^1^ Management & Business Administration Aliah University Kolkata India; ^2^ School of Fashion, The Creative School Toronto Metropolitan University Toronto Ontario Canada; ^3^ Sustainability Competence Centre Széchenyi Istvàn University Győr Hungary

**Keywords:** halal cosmetics, PRISMA, religiosity, systematic literature review, TCCM

## Abstract

**Background:**

The COVID‐19 epidemic has fuelled increasing anxiety regarding the environment and religiosity. Moreover, the worldwide halal cosmetics industry is expected to experience significant growth in the years ahead. Still, the reasons that drove people all across to choose halal cosmetics are unclear.

**Objectives:**

The study aims to find factors influencing halal cosmetics purchases. Based on the findings, the review proposes a conceptual framework and new directions for future research in the context of halal cosmetics.

**Method:**

The PRISMA & TCCM framework systematically evaluates 51 empirical articles on consumers' behaviors regarding the purchase of halal cosmetics from Scopus between 2014 and 2024 for the review.

**Results:**

The review suggests forthcoming investigations to utilize Consumer Culture theory, Social Practice theory, and the UTAUT model. By highlighting patterns in halal cosmetics literature, the paper helps to guide future research in underexplored domains such as artificial intelligence and e‐commerce.

**Practical Implications:**

The review contributes to the existing corpus of knowledge regarding the theoretical perspective of contemporary halal marketing through its proposed conceptual framework. In particular, scholars, academicians, and practitioners may delve into the reliable body of literature on halal cosmetics.

**Originality/Value:**

This study examines consumer behavior regarding the consumption of halal cosmetics. It pinpoints research gaps and offers future avenues using the TCCM framework. In addition, it provides the conceptual framework for measuring the behavior of halal cosmetics.

## 1 Introduction

The word “halal” originates from “Islam” and refers to anything that is lawful under the Islamic religion [1]. To be considered halal, both the ingredients and the manufacturing process must follow the guidelines of Islamic law. Thus, Muslims all around the world seek authentic halal offerings that follow a protocol consistent with Islamic practice or faith [2]. Given the significance of halal certification, marketing strategies have evolved to cater to the needs and preferences of Muslim consumers. “Halal marketing” and “Islamic marketing” are intrinsically associated with “ethnic marketing” [3, 4]. The novelty of these strategies, therefore, is defined by their link to religion [5]. Islamic marketing and halal marketing guide and provide directions or instructions on how to live and behave in a particular manner in everyday life. Contemporary culture places an emphasis on attractiveness and personal style [6], and cosmetics have become an increasingly important part of people's lives [7]. The cosmetics market is growing faster than other product segments globally and is projected to register further growth in the coming years [8]. Given the demand for cosmetics products, Muslim consumers around the world look for products that are Sharia‐compliant or halal‐certified. They hold mistrust toward non‐halal cosmetics brands regarding Sharia compliance. Non‐halal cosmetics are cosmetics that do not adhere to the Sharia principles of quality, safety, and ingredients. Most non‐halal cosmetics contain substances like alcohol, collagen, gelatin, and lactic acid that are not in accordance with the Sharia principle. Therefore, halal cosmetics ensure that they are made according to Sharia principles and also ensure product quality and safety for their users [9].

When it comes to business, it is evident that the worldwide Muslim population is growing and presenting a significant potential for the global Islamic economy. This growth is comparable to that of China and India, already established as major growth markets [10]. There has been a noticeable improvement in the socioeconomic status of Muslim households, with rising disposable incomes and a resulting increase in purchasing power. It is worth noting that Muslims are emerging as a formidable economic force, and there is increased demand for halal cosmetics products that have not been seen before [11]. Due to the increased demand, companies are increasingly recognizing Muslim‐majority markets as lucrative [12]. The COVID‐19 pandemic has changed the shopping habits of the younger generation, making them more environmentally conscious [13]. As a result, they are more inclined to pay a premium price for sustainable products. The pandemic has prompted religious dedication globally [14]. Furthermore, the market is driven by religiosity, which is a primary driver of halal product offerings. As Fortune Business Insight (2025) forecasts, the halal cosmetics market size stands at $53.12 billion in 2025 [15] and is anticipated to be $115.03 billion in 2032, representing an annual growth rate of 11.67% [16].

The previous review study by Nordin et al. [17] was based on only skin whitening or lightening cosmetics, limiting its scope and generalisability. Another review study by Isa et al. [18] was based on a review of 14 papers only. In addition, the framework employed was the S‐O‐R framework, which gives a different methodological view. There was a restriction of the year 2016–2021 for the study. However, this review has a wider coverage in terms of the year (2014–2024) with no restriction or filter on the year. However, due to a review sample of 14 papers merely, Isa et al.'s [18] findings are more inclined toward product characteristics. Hence, this review has an adequate pool of papers (51) utilizing the TCCM framework, which no study has employed so far. The main value addition of this research is to provide a conceptual structure, which is the most significant characteristic and purpose of a literature review, which no other review has provided. The evaluation through this review addresses the factors in play when purchasing halal cosmetics. As a result, the study develops two research questions:

**RQ1:** What are the most crucial “theories,” “context,” “characteristics,” and “methods” in the current scholarly works of halal cosmetics?

**RQ2:** What are the future study avenues related to consumer behavior on halal cosmetics?

## 2 Methodology

**FIGURE 1:**
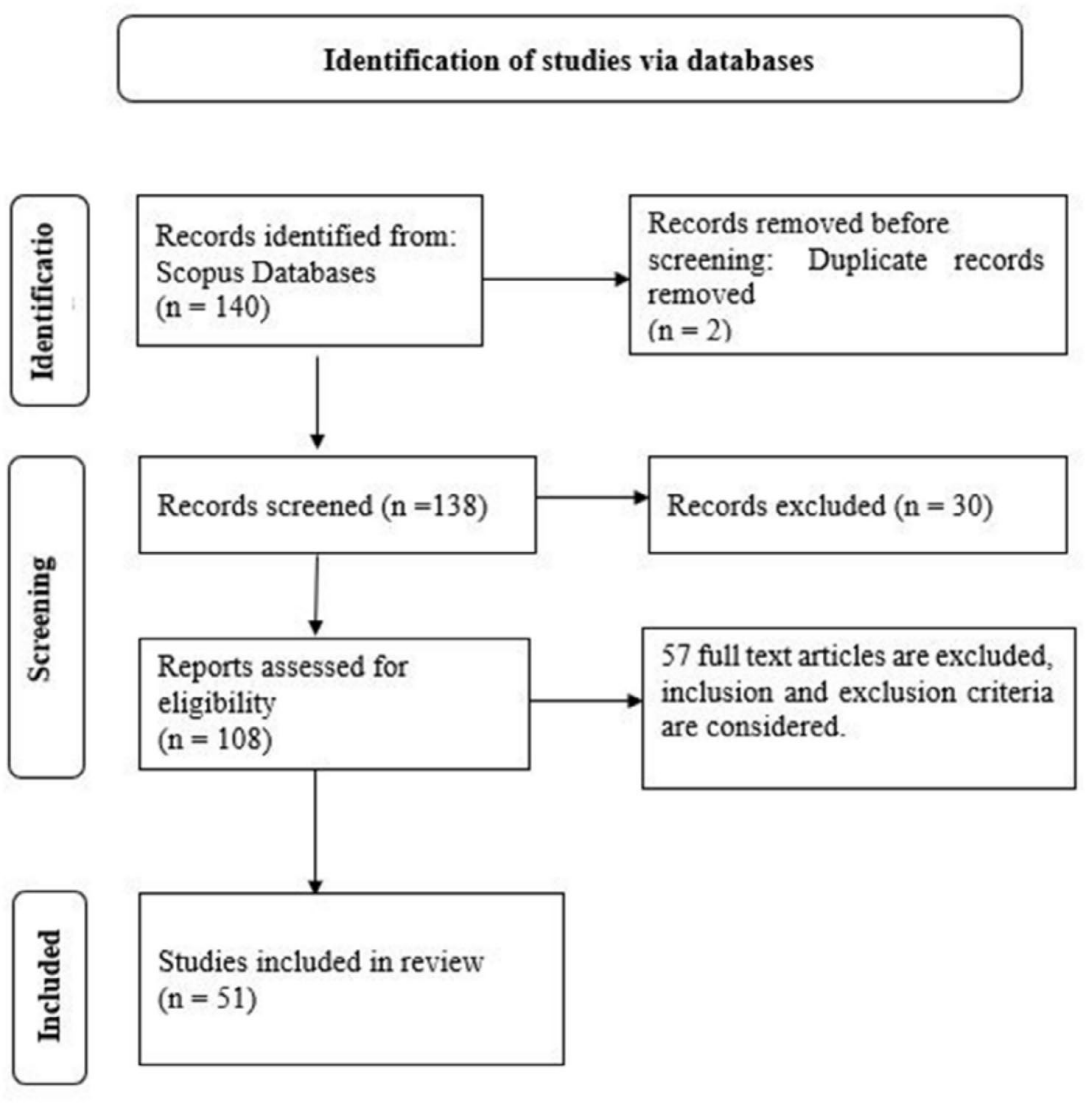
PRISMA framework for article extraction. *Source:* Page et al. [22].

The study employed a range of search terms to extract halal cosmetic adoption literature. The study adheres to the literature review guidelines outlined by Jain et al. [19], Rasheed et al. [20], and Uddin et al. [21]. The review incorporates consumer psychological articles on halal cosmetics from the business and humanities domains, using the “Preferred Reporting Items for Systematic Review and Meta‐Analysis Protocols” (PRISMA) technique (Figure 1). The search terms used were “Halal Cosmetic*” OR “Muslim Cosmetic*” OR “Halal Facial Care” OR “Halal Skincare*” OR “Halal Skin care” OR “Muslim Skincare” OR “Halal Personal care” OR “Muslim Personal care*”. The search terms above were used to retrieve academic papers by matching them to the titles and abstracts in the Scopus database, resulting in 140 research papers. However, after the application of the inclusion and exclusion criteria, a total of 108 publications were identified. The inclusion criteria included empirical articles and conference papers from reputed publishers. Reviews and book chapters were excluded. Further to the selection criteria, the review included management‐related subjects and other social science subjects with a focus on consumer behavior, ensuring the relevance of the included studies. As a result, a total of 51 studies were included for review. After conducting a content check, we found that the studies selected for review ranged from 2014 to 2024. The corpus of papers (51) demonstrates that the field is well‐established for a literature review, following the guidelines of Paul and Criado [23]. Subsequently, extracted papers were utilized for a structured evaluation of scholarly works based on the “Theory‐Context‐Characteristics‐Methodology” (TCCM) protocol [24].

## 3 Results

### 3.1 Publication Trend

**FIGURE 2:**
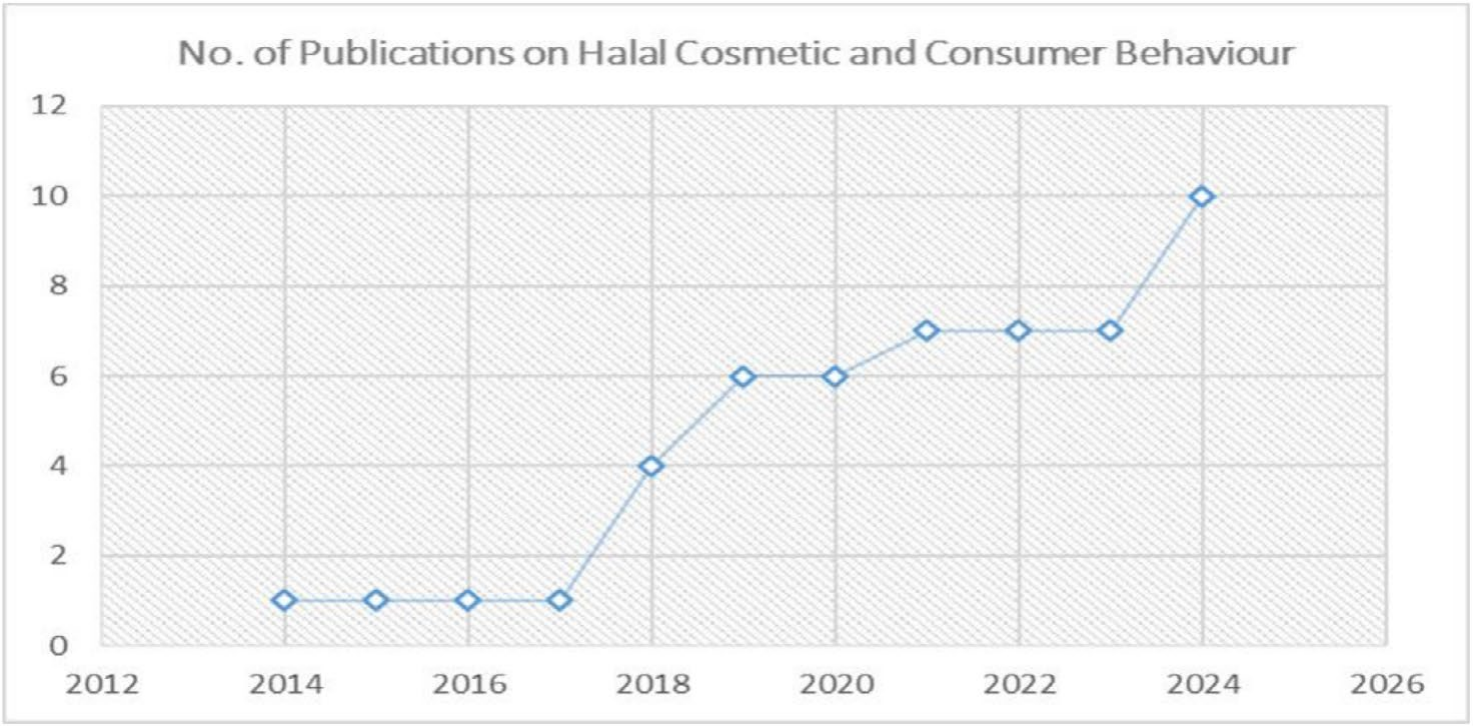
Trends in the publication related to consumers' halal cosmetic behavior. *Source:* Scopus.

After 2014, the research focusing on the influential factors for halal cosmetics purchases became prominent. According to the search results from the Scopus database, the publications on this topic have increased since 2014 (see Figure 2). The rise in publications, from just four articles between 2014 and 2017 to 47 articles between 2018 and 2024, clearly indicates a growing awareness of halal products among consumers. Moreover, the shift in trend toward eco‐conscious products and increased religious fervor could be attributed to the COVID‐19 pandemic [14]. Thus, to cater to these trends, research on halal cosmetics has increased.

### 3.2 TCCM Analysis

The TCCM framework uses theory, context, characteristics, and methodology parameters in this review to extract gaps in the halal cosmetics literature, and it is one of the most reliable frameworks in the consumer behavior context [24].

#### 3.2.1 Theories

##### 3.2.1.1 Theory of Reasoned Action

Regarding consumer behavior, TRA and TPB are the most commonly used theories. Eight studies from the corpus utilized the TRA theory. Briliana and Mursito [25] and Widyanto and Sitohang [26] highlighted the importance of knowledge, subjective norms, and religiosity in driving the purchase of halal cosmetics. Similarly, Sudarsono and Nugrohowati's [27], Nuryakin et al. [28] and Suryadi et al. [29] findings stressed the importance of knowledge and religiosity [29]. Khalid et al. [30] study suggested subjective norms, attitude, and product positioning significantly influence purchase intention toward halal cosmetics. In contrast, Rahman et al. [31] found knowledge ineffective in influencing the purchase intent. Sama and Trivedi [32] focus on core TRA variables like subjective norms and beliefs that affect attitude and brand love, which in turn influence loyalty.

In summary, the studies that used TRA theory, religion, knowledge, and halal certification can create an opinion in the minds of Muslim consumers, further shaping their attitude toward halal cosmetics. The subjective norm in this context is social media, a virtual environment that can shape their intention and behavior.

##### 3.2.1.2 Theory of Planned Behavior

The TPB theory is the extended model of TRA. Perceived behavioral control is the added variable in this theory. The benefit of the theory lies in its contextual addition of variables [33]. Ngah et al. [34] and Widiastuti et al. [35] emphasized that attitude, brand image, and subjective norms influence purchase intent. However, Ngah et al. [34] further highlighted that male behavior is not influenced by subjective norms; females are affected by what society thinks of them, implying that subjective norms are an influencing variable. Further, the male looks at ease in the use (perceived behavioral control) of halal cosmetics, whereas perceived behavioral control (PBC) has no impact on females. PBC has also been reported as a non‐influencing factor by Bhutto et al. [36]. Similarly, Haque et al. [37] found perceived behavior, attitude, and religiosity as key drivers, but subjective norms were found to have no effect in the context of halal cosmetics. Khan et al. [38] study states that variables like ingredient safety, halal logo, and advertisement are influential, whereas religiosity has a minimal impact. In addition, Handriana et al. [8] found that “perceived value,” “religious beliefs,” and “brand image” influence attitude. Lastly, Irfany et al. [39] noted that “environmental knowledge” and “religiosity” influence “attitude,” which in turn influences purchase intent. Additionally, “halal logos” and “eco‐labels” were discovered as crucial variables to increase awareness.

In general, the TPB model research produced inconsistent and conflicting findings, particularly regarding religiosity. Religion was shown to be significant when used as an attitude predictor, but it had no effect when used as a measure of perceived behavioral control. The PBC did not have any major influence on purchase intention for halal cosmetics, whereas subjective norms played a crucial role in influencing intent.

##### 3.2.1.3 Stimulus Organism Response

The study by Suparno [40] found religiosity and hedonic value as the stimulus variables for affecting cognitive and affective attitudes, which further influence online purchase intent. Ngah et al. [9] found celebrity influence affects attitude and brand image. However, religiosity tends to make consumers regular users of halal products. Therefore, marketers should focus on religiosity, hedonic values, and celebrity endorsement.

##### 3.2.1.4 Cognitive Fit Theory and Elaboration Likelihood Model Theory

Consumers are more involved in the product and information when they are motivated and interested. When they are not interested or do not have the capacity to be involved with the product, they look for simple reasons or shortcuts to make decisions regarding products. If the consumers are interested in halal cosmetics and are able to understand online reviews (e‐Wom), they will go through the comments to check the authenticity and consistency. Further, the consumer will find whether these comments are inclined toward positivity or negativity (Valence). This type of indulgence takes more involvement and effort [7]. The e‐Wom has a significant role in influencing attitude, subjective norms, and perceived behavioral control [41], but e‐Wom cannot directly influence purchase intention [42].

But in case consumers are not much interested or motivated by the product, the Elaboration Likelihood Model guides them to form their attitudes. If consumers are not motivated regarding halal cosmetics, they will not go for details about the product online. They won't focus on the quality and consistency of reviews. Consumers will look for a greater number of reviews for a product. These consumers use peripheral routes for decision‐making. Therefore, factors such as valence, quality, and consistency will impact attitude only when a person is motivated and looks for involvement in a product.

##### 3.2.1.5 Theory of Consumption Value

Huriah et al. [43] found that if consumers or people have knowledge and are religious, they will form a positive attitude toward halal cosmetics. A positive attitude will further lead to actual purchases. The study also delves into the TCV variable to find out the reason for selecting halal cosmetics. Curiosity (epistemic value) and how the product generates emotions (emotion value) are the main factors influencing halal cosmetics purchases. Similarly, Rachman and Amarullah [44] found emotional value, epistemic value, and functional value to be influencing factors for attitude and repurchase intention. Surprisingly, Yeo et al. [45] found that in the context of halal cosmetics, the product usefulness (functional value) and desire to try new things (epistemic value) do not impact consumers' intent or attitude toward halal cosmetics.

##### 3.2.1.6 Diffusion of Innovation Theory

Mohezar's [66] study used the Diffusion of Innovation theory and found that features of the product, how people perceive the product (social influence), how people try new things (individual innovativeness), and how religious they are. Similarly, Ali et al. [47] confirm the role of social influence, religiosity, and product qualities in influencing halal cosmetic purchases. In addition, product awareness and price are also important factors in determining attitudes toward halal cosmetics.

#### 3.2.2 Characteristics

##### 3.2.2.1 Marketing‐Related Factors

The origin of the product tends to play an important role in halal cosmetics. The scholarly work by Mahri et al. [48] found that country of origin (COO) has a strong impact on influencing purchase decisions in Indonesia. In addition, people are more inclined to buy imported halal cosmetics. They believe imported halal cosmetics are authentic and reliable [49]. However, in contrast, the study by Hong and Kamaruddin [50] highlights that neither the COO nor the halal logo influences buying decisions. The factors that matter are the education level, gender, and inclination toward local products. Zaidi et al. [51] discussed the issue of knockoff cosmetics. It stressed that “age,” “income,” and “brand consciousness” affect how consumers perceive counterfeit cosmetics. Overall, the findings imply that one market strategy is not enough for every market. A local market strategy is required before making a marketing decision.

The main factors explaining the purchase intention toward halal cosmetics include bloggers, Arabic names, religiosity, and halal logos. These factors explain about 80% of the consumers' purchase intention, as per Putri and Abdinagoro [52]. Among these factors, the halal logo has the strongest influence on the intention, followed by celebrity endorsement, according to a study by Putri et al. [53]. The study by Sudaryanto [54] and Ishak et al. [55] highlighted that consumers are influenced by brand effectiveness, quality of the product, and celebrity endorsement. In addition to the trust of the consumers in the quality of the product [56], ingredients are one crucial factor [57]. The study by Islam [58] showed people's concern toward ingredients like animal fat from haram sources and alcohol, and finally, the skepticism toward products from a country that is the non‐Muslim majority. Meanwhile, Hong et al. [59] found religious faith, ingredient concerns, pricing, availability, the halal logo, and recommendations from social groups to be influencing factors. Halal marketing activities need to be increased, as they also influence purchase intention [60]. Point‐of‐sale marketing materials such as free samples, coupons, interactive kiosks, and QR Codes will influence purchase intent among consumers [61].

##### 3.2.2.2 Psychological Variables

Psychological factors like feeling and associating with products affect decision‐making. Osman et al. [62] found that for adults, the positive and negative feelings toward halal cosmetics influence intention. In addition, Adiba [63] highlighted that as foundational variables, knowledge and awareness are important for other factors to play any influencing roles. Interestingly, Septiarini et al. [64] found that the “halal logo,” “awareness,” and “positive halal brand image” can shape the attitude of non‐Muslim consumers. The findings imply marketers should highlight universal values like purity, safety, and quality to strengthen brand image since even non‐Muslims respond well to halal branding.

#### 3.2.3 Context

Halal cosmetics literature encompasses many contexts, including nations, industries, and platforms.

##### 3.2.3.1 Country

Among the 51 studies on halal cosmetics, all of them were based on Asian nations. The trend could be due to the majority of the Muslim populace in Asia and the importance of halal cosmetics and products in these regions. Indonesia (22) and Malaysia have (17) studies, comprising about 80% of approx. studies. Other major economies, like India, have four studies, whereas China and Pakistan have three studies each. Bangladesh and Saudi Arabia have two and one study, respectively. The concentration of studies in Malaysia and Indonesia shows a big market potential for cosmetic companies. The findings call for a global understanding of halal cosmetics, especially in Muslim‐minority markets.

##### 3.2.3.2 Industry

The research on halal cosmetics so far is mainly on offline or traditional shopping experiences (84% approx, 43 papers). Only a small part of the research (16%) looks at how consumers buy halal cosmetics online. Although online shopping is booming in the skincare segment, the literature available related to it is limited.

#### 3.2.4 Method

The review highlights the striking pattern in methodological choices in the literature related to halal cosmetics. The majority of the studies (49) employed a quantitative approach, which relied on structured surveys and statistical analyses to understand consumer behavior, whereas only two studies, namely Shahid et al. [65] and Musa [66], adopted a qualitative methodology to uncover motivations and intentions toward halal cosmetics. The qualitative methodology relies on an unstructured pattern of interviews or focus group discussions, which can offer a contextualized understanding of consumer attitudes and experiences.

The researcher's quantitative and statistical inclination shows their most employed tool called structural equation modeling (SEM). 74% of studies (38) used the SEM tool due to its effectiveness in measuring the association between independent and dependent variables [67]. This tool helps to measure how awareness might influence attitude and further purchase intent. 12 studies utilized covariance‐based SEM, which is an effective tool in testing theories and confirming how well variables in a framework fit together. The studies utilized software like AMOS, LISREL, and MLR to perform SEM.

Surprisingly, variance‐based SEM (PLS‐SEM) was utilized by the majority of studies (26) as these methods are utilized when the conceptual framework is complex, or the sample size is smaller. The other methods employed for finding results comprised 10 papers. Among these, four employed multiple linear regression. Other methods adopted by studies include correlation analysis, ANOVA, Choice Modeling, and group analysis. The dominance of SEM as a methodological approach highlights the maturity of theory development related to halal cosmetics (Table 1).

**TABLE 1:** Articles included in the study.

S.no	Authors	Statistical methods	Sample population—gender	Country of study
1	Nuryakin et al. [28]	CB SEM	Female	Malaysia & Indonesia
2	Widiastuti et al. [35]	PLS SEM	Male and female	Indonesia
3	Hasan et al. [60]	PLS SEM	Female	Bangladesh
4	Fiandari et al. [42]	PLS SEM	Female	Indonesia
5	Hussain et al. [56]	PLS SEM	Female	Pakistan
6	Rachman and Amarullah [44]	PLS SEM	Male and female	Indonesia
7	Mohd Shelahudin et al. [68]	PLS SEM	Female	Malaysia
8	Bhutto et al. [41]	PLS SEM	Male and female	Pakistan
9	Virgiawan et al. [61]	PLS SEM	Female	Indonesia
10	Shmailan and Alfalih [69]	ANOVA	Male and Female	Saudi Arab
11	Bhutto et al. [36]	CB SEM	Male and female	Pakistan
12	Septiarini et al. [70]	CB SEM	Male and female	Malaysia, Indonesia & Singapore
13	Irfany et al. [39]	PLS SEM	Male and female	Indonesia
14	Shahid et al. [70]	CB SEM	Female	India
15	Mahri et al. [48]	PLS SEM	Male and female	Indonesia
16	Al‐Banna and Jannah, [71]	PLS SEM	Female	Indonesia
17	Zaidi et al. [51]	Anova & multiple regression	Male and female	Malaysia
18	Huriah et al. [43].	PLS SEM	Male and female	Indonesia
19	Osman et al. [62]	PLS SEM	Male and female	Malaysia
20	Islam, [58]	Chi square test & binary logistic regression	Male and female	Bangladesh
21	Sudaryanto et al. [54]	Multiple linear regression	Female	Indonesia
22	Ngah et al. [9]	PLS SEM	Male and female	Malaysia
23	Widyanto and Sitohang [26]	PLS SEM	Male and female	Indonesia
24	Yulianto et al. [72]	CB SEM	Male and female	Indonesia
25	Ngah et al. [34]	PLS SEM	Male and female	Malaysia
26	Abd Jalil et al. [73]	PLS SEM	Male and female	Malaysia
27	Khalid et al. [30]	PLS SEM	Male and female	Malaysia
28	Handriana et al. [8]	CB SEM	Male and female	Indonesia
29	Khan et al. [38]	PLS SEM	Male and female	Malaysia
30	Suhartanto et al. [57]	PLS SEM	Female	Indonesia
31	Anubha [7]	CB SEM	Female	India
32	Hong et al. [49]	Choice modeling, conjoint analysis and logit model	Male and female	China
33	Hong and Kamaruddin [50]	Logit Model	Male and female	China
34	Sudarsono and Nugrohowati [27]	CB SEM	Male and female	Indonesia
35	Ishak et al. [55]	*T*‐test and correlation analysis	Female	Malaysia
36	Suryadi et al. [29]	PLS SEM	Female	Indonesia
37	Suparno [40]	CB SEM	Female	Indonesia
38	Putri et al. [53]	Correlation analysis	Male and female	Indonesia
39	Arbak et al. [74]	Pearson correlation and multiple regression	Female	Malaysia
40	Adiba [63]	PLS SEM	Female	Indonesia
41	Ali et al. [47]	PLS SEM	Male and female	Malaysia
42	Sama and Trivedi [32]	CB SEM	Male and female	India
43	Hong et al. [59]	Logit model	Male and female	China
44	Putri and Abdinagoro [52]	PLS SEM	Female	Indonesia
45	Yeo et al. [45]	CB SEM	Female	Malaysia
46	Haque et al. [37]	CB SEM	Male and female	Malaysia
47	Shahid et al. [65]	Focus group & in‐depth interviews	Male and female	India
48	Briliana and Mursito [25]	PLS SEM	Female	Indonesia
49	Mohezar et al. [66]	PLS SEM	Male and female	Malaysia
50	Rahman et al. [31]	Paired sample *t*‐test	Male and female	Malaysia
51	Musa [66]	Focus group analysis	Female	Malaysia

## 4 Proposed Conceptual Framework

After analyzing the factors and theories about halal cosmetics, a framework grounded on the TPB and TRA is proposed. The review study finds factors such as halal label, halal awareness, religiosity, knowledge, vloggers' involvement, and eco‐consciousness [26, 36, 38, 39, 48, 51, 70]. Religiosity, eco‐friendliness, and celebrity vloggers' involvement are related to social expectations in society, thus replacing the subjective norm variable. The perceived behavioral control factors include awareness, knowledge, and halal labels. These variables will reduce the difficulty of identifying and choosing halal‐compliant cosmetics, enhancing the consumer's sense of control over their purchasing decisions.

In this proposed model, the attitude variable acts as a mediator between independent variables and purchase intention. Attitude plays a significant role in influencing purchase intention, but the independent variables have not been able to influence consumers' purchase intention. Therefore, in that case, the strength of these independent variables depends on their ability to influence consumer attitude. Given this instance, the study proposes attitude as a mediator. To make the framework relevant to the current technological innovation and issues related to consciousness not getting converted to final purchase. The role of AI in the website is incorporated based on the study by Yulianto et al. [72].

## 5 Future Research Direction

The TCCM framework highlights the future research avenues and provides a gap in the present literature, which is discussed below:

### 5.1 Theory

TPB and TRA models have been extensively utilized in the literature. Many studies in halal cosmetic literature lack a theoretical framework. The UTAUT model can predict the dynamic online environment in relation to halal cosmetics. The expectations, enabling factors, and social angle of the UTAUT theories with extended variables may predict purchase intention. Future studies may incorporate Consumer Culture theory, which could explore how social identity and culture drive consumption related to cosmetics. Additionally, social practice theory and dual process theories could be explored. The Social Practice theory would highlight consumption as a socially shared practice among Muslims rather than individual attention, whereas the dual process theory would be able to measure whether the purchase related to halal cosmetics is impulsive or a thoughtful and rational purchase.

### 5.2 Context

Despite the sizeable Muslim population living in India, Pakistan, and Bangladesh, fewer studies have focused on halal cosmetics in these economies. The literature focuses mostly on Indonesia and Malaysia. There is a clear geographical context gap that leaves a void in nations like India, Pakistan, and Bangladesh, as well as in halal‐conscious economies like the UK, UAE, and various African nations. The studies in these nations will balance the generalizability of the findings. The African and European contexts require more studies. Additionally, due to rapid e‐commerce usage and digital consumption, most research still focuses on offline stores. Future investigations should explore online purchase behavior. The majority of studies focus on female consumers, neglecting the growing market segment of male grooming and cosmetics.

### 5.3 Characteristics

As the sustainability concern is increasing, it is important to understand and highlight the eco‐friendly attributes of halal cosmetics. The eco‐friendly nature would widen the market and acceptance among consumers. In parallel, brand equity is a major driver of consumer behavior [75]. In the halal cosmetic context, whether the halal marketing initiative is adding to the brand equity and influencing purchase intent is under examined. Finally, the role of AI‐powered e‐commerce websites, recommendation systems, and digital marketing in engagement and purchase behavior in the halal cosmetic segment is the future avenue for research.

### 5.4 Methods

The current methodological landscape remains inclined toward quantitative approaches. More than 90% of studies in the literature adopted qualitative statistical techniques. This one‐sided dominance of quantitative paves the way for qualitative study in the future. The researchers should adopt qualitative research, such as “in‐depth interviews,” “focus group discussions,” and “ethnographic studies,” to uncover consumer motivation and preferences for halal cosmetics in a more genuine way. Apart from the traditional approach through a mixed‐method approach and triangulation, the integration of sentiment analysis, topic modeling, and machine learning‐based pattern detection can reveal hidden trends that are overlooked through conventional surveys. The AI‐driven insights through topic modeling and sentiment analysis would lead to a predictive model in relation to consumer behavior and, therefore, will aid in better product development and market positioning by companies.

## 6 Discussion and Conclusion

This review has conducted a thorough examination of the “TPB,” the TRA, the TAM, the SOR framework, and the DOI theories, alongside various factors pertaining to “brand,” “product,” and “demographic” influence inclination toward halal cosmetics. The literature identifies several significant factors determining purchase intention for halal cosmetics, including “subjective norms,” “attitude,” “perceived behavioral control,” “self‐efficacy,” and “knowledge.” Research employing the “TPB” and the extended “TRA” as foundational frameworks identified these factors as relevant. It was discovered that attitude serves as a crucial mediating connection among various exogenous variables such as “religiosity,” “awareness,” “commitment,” and others. Moreover, research employing SOR as its theoretical framework has recognized “hedonic value,” “utilitarian value,” “perceived value,” “religious belief,” and “environmental knowledge” as significant stimuli for forecasting whether or not consumers would buy or use halal cosmetics. The inclination to purchase is similarly shaped by “emotional,” “epistemic,” and “conditional value,” according to research grounded in TCV theory. The “perceived attributes of innovation,” “individual innovativeness,” and “product characteristics” emerged as significant variables within the framework of DOI theory concerning halal cosmetics. The attributes of a product, including the “halal certification,” its provenance, whether it is imported, as well as the quality and composition of ingredients utilized in cosmetic formulations, significantly impact consumer choices regarding halal cosmetics. Within the realm of demographic considerations, the variables of “education,” “gender,” and “age cohort” serve as significant indicators of halal cosmetic practices. Moreover, the TCCM approach underscores the shortcomings of existing research. The application of Social Practice theory, Consumer Culture theory, and the UTAUT model is proposed as a means to assess the factors that influence the adoption of halal cosmetics.

In contrast, a study conducted by Annabi and Ibidapo‐Obe [76] in the United Kingdom found that cosmetics manufacturers are not following the product standards related to halal compliance. Given the circumstances, the AI‐powered website will address their concern about halal compliance. The AI aims to eliminate barriers to purchases by establishing a standard to address counterfeit halal cosmetics. The more aware a person is, the more inclined he will be to use halal cosmetics. However, the positive or negative aspect of awareness among non‐Muslims regarding halal products remains the context of further study.

## 7 Implications

**FIGURE 3:**
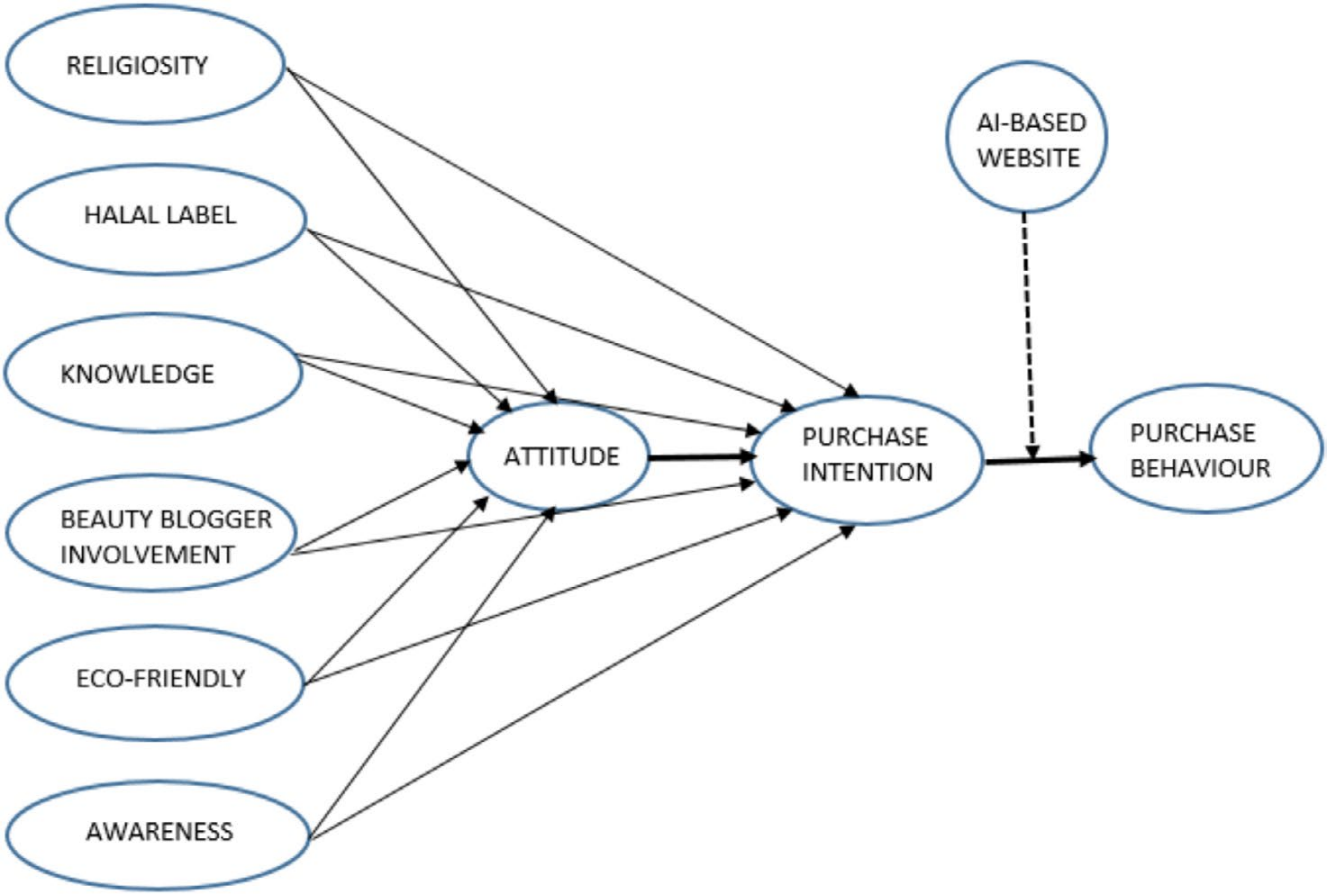
Conceptual framework—Drivers of halal cosmetics adoption. *Source:* Concept by authors.

This study added to the existing structure of knowledge regarding the theoretical perspective of contemporary halal marketing through its proposed conceptual framework (Figure 3). In particular, scholars, academicians, and practitioners may delve into the reliable body of literature on halal cosmetics. This would yield valuable insights pertaining to consumer behavior factors. Moreover, the findings of this review could potentially be applied to studies investigating consumer behavior in other product categories. Interestingly, the study will enhance awareness among consumers pertaining to halal products. The findings suggest that since attitude is a mediator across most studies, campaigns focusing on creating positive emotional and rational associations are required. The campaigns should communicate ethical, religious, environmental, and quality aspects of halal cosmetics. The influence of subjective norms on purchase is evident. The social factor could be harnessed by endorsing halal cosmetics by prominent community leaders or beauty influencers. The marketers need to capitalize on the hedonic and utilitarian values. The product design providing the dual benefit of spirituality and trendiness is suggested for halal cosmetics. Lastly, it is observed that the Muslim population across different economies needs to be catered to with different marketing strategies due to vast differences in choices and consumption behavior.

## Author Contributions

Furquan Uddin performed the PRISMA method and data extraction with the help of Md Billal Hossain. Md Wasim Raza designed the structure of the manuscript and gave final approval of the version of the manuscript. Furquan Uddin and Osmud Rahman worked together on the technical details. Md Wasim Raza worked on the original draft preparation, reviewed, and edited the article. Furquan Uddin and Md Billal Hossain also act as corresponding authors of this article and are responsible for editing the paper based on editors' and reviewers' comments. The supervision and funding acquisition is made by Md Billal Hossain.

## Ethics Statement

The authors have nothing to report.

## Conflicts of Interest

The authors declare no conflicts of interest.

## Data Availability

The data has been extracted using the Scopus database, which is subject to the license. The data can be downloaded, replicating the process mentioned in the methodology.
